# Mucolipin TRP Cation Channel 1 Depletion Promotes Gastric Cancer Cell Ferroptosis Through the AKT Pathway

**DOI:** 10.1002/kjm2.70301

**Published:** 2026-10-31

**Authors:** Jing Wang, Jun‐Chen Ge, Jian Ma, Xin Wang, Wen Jia, Li Zhu

**Affiliations:** ^1^ Department of Gastroenterology The 962th Hospital of the Chinese People's Liberation Army Harbin China; ^2^ Department of Endocrinology, First Affiliated Hospital Heilongjiang University of Chinese Medicine Harbin China; ^3^ The First Clinical Medical College Nanchang University Nanchang China; ^4^ Department of Endoscopy General Hospital of Northern Theater Command Shenyang China

**Keywords:** AKT pathway, ferroptosis, gastric cancer, MCOLN1/TRPML1, TRPC1 and TPCN1

## Abstract

Mucolipin TRP cation channel 1 (MCOLN1), also called TRPML1, drives the progression of various cancers and inhibits ferroptosis in a number of diseases. This study aimed to investigate whether MCOLN1 depletion regulates ferroptosis in gastric cancer and its molecular regulatory crosstalk with the AKT pathway. MCOLN1 siRNA was transferred into AGS and HGC27 cells to deplete MCOLN1 expression. SC79 was added to activate the AKT pathway. The relationship between MCOLN1 and AKT was analyzed via public databases. MCOLN1 depletion reduced glutathione (GSH) but increased malondialdehyde (MDA), reactive oxygen species (ROS), and Fe^2+^ levels in both cell lines. MCOLN1 depletion also decreased SLC7A11 expression and increased ACSL4 expression in HGC27 cells but only downregulated SLC7A11 expression in AGS cells. Cell viability decreased after MCOLN1 depletion in both cell lines. The protein–protein interaction network showed potential associations among MCOLN1, TRPC1, TPCN1, and AKT1, with TRPC1 and TPCN1 positioned between MCOLN1 and AKT1 in the network; online public database analysis revealed that MCOLN1 was positively correlated with TRPC1, TPCN1, and AKT1 in gastric cancer tissue samples. Additionally, MCOLN1 depletion decreased p‐AKT expression in both cell lines. Next, SC79 effectively activated the AKT pathway in both cell lines and suppressed ferroptosis. Moreover, AKT activation attenuated the ferroptosis‐promoting effect of MCOLN1 depletion in both cell lines. In conclusion, MCOLN1 depletion promotes gastric cancer cell ferroptosis, at least in part, through inhibition of the AKT pathway. TRPC1 and TPCN1 may be involved in this regulatory axis, but their precise roles remain to be determined.

## 1 Introduction

Gastric cancer is among the most frequently diagnosed malignancies of the digestive tract and is characterized by intricate pathogenic mechanisms and significant molecular heterogeneity, which contribute to varied outcomes and generally unfavorable prognoses [1]. Ferroptosis, a recently identified form of regulated cell death driven by intracellular iron accumulation and lipid peroxidation, plays a key role in the development of gastric cancer [2]. A growing body of comprehensive reviews indicates that ferroptosis can inhibit gastric cancer progression, affecting tumor growth, metastasis, epithelial–mesenchymal transition, and resistance to treatment [2, 3, 4]. Moreover, genes associated with ferroptosis hold promise as prognostic indicators for gastric cancer [5]. Thus, investigating the mechanisms that induce ferroptosis in gastric cancer is highly important.

Mucolipin TRP cation channel 1 (MCOLN1), also known as transient receptor potential channel mucolipin 1 (TRPML1), is a non‐selective cation channel found on late endosomes and lysosomes. It helps release lysosomal contents into the cytoplasm and plays key roles in autophagy, exocytosis, and endocytosis [6]. Previous work has linked MCOLN1 to tumor progression in several cancer types, including triple‐negative breast cancer, melanoma, lung cancer, and hepatocellular carcinoma [7, 8, 9, 10]. Research on its role in gastric cancer is still limited, but one study revealed that MCOLN1 is upregulated in gastric cancer patients and that high expression of MCOLN1 is correlated with poor prognosis; mechanistically, MCOLN1 drives the lysosomal exocytosis of galectin‐1 (LGALS1) and insulin‐like growth factor binding protein 7 (IGFBP7), which subsequently activates the AKT pathway to promote gastric cancer progression [11]. Interestingly, other evidence has shown that depleting MCOLN1 in breast cancer reduces tumor growth and stemness while increasing chemosensitivity by promoting ferroptosis [12]. Moreover, TRPML1 overexpression has been reported to confer stronger ferroptosis resistance in cancers with hyperactive AKT signaling [13]. Overall, we hypothesized that MCOLN1 depletion might trigger ferroptosis in gastric cancer through AKT inactivation, although direct evidence remains scarce.

This study aimed to characterize how MCOLN1 depletion regulates ferroptosis in gastric cancer and its molecular regulatory crosstalk with the AKT pathway.

## 2 Materials and Methods

### 2.1 Cell Culture

Two human gastric cancer cell lines, AGS and HGC27 (both from Cellverse, China), were used in this study. AGS cells were grown in Ham's F‐12K medium (Servicebio, China), while HGC27 cells were cultured in Dulbecco's modified Eagle's medium (Servicebio, China). Both media contained 10% fetal bovine serum (Procell, China) and 1% penicillin/streptomycin (Servicebio, China). The cells were incubated at 37°C in a 5% CO_2_ atmosphere.

### 2.2 Transfection and SC79 Intervention

Negative control small interfering RNA (siRNA) and MCOLN1‐specific siRNA were designed and synthesized by Shanghai GenePharma Co. Ltd. (China). For transfection, each siRNA was mixed with GP‐transfect‐Mate (Genepharma, China) to form complexes following the manufacturer's protocol. AGS and HGC27 cells were then incubated with the complexes. The cells were divided into three groups: blank (no transfection), siNC (transfected with negative control siRNA), and siMCOLN1 (transfected with MCOLN1 siRNA). Further assays were carried out 48 h after transfection.

For the SC79 intervention experiments, the AKT activator SC79 (Yuanye Bio, China) was added to AGS and HGC27 cells at 24 h post‐transfection at a final concentration of 20 μM as described previously [14]. The treatment lasted another 24 h. Cells were then assigned to four groups: siNC (negative control siRNA only), siMCOLN1 (MCOLN1 siRNA only), siNC + SC79 (negative control siRNA plus SC79), and siMCOLN1 + SC79 (MCOLN1 siRNA plus SC79). All subsequent measurements were performed at 48 h after the initial transfection.

### 2.3 MCOLN1 Overexpression and Erastin (Era) Intervention

An MCOLN1 overexpression plasmid (oeMCOLN1) and a negative control plasmid (oeNC) were obtained from Shanghai GenePharma Co. Ltd. AGS and HGC27 cells were transfected with oeMCOLN1 or oeNC using GP‐transfect‐Mate. After 24 h of transfection, Era (10 μM, MedChemExpress, China) was added to induce ferroptosis. The cells were divided into four groups: oeNC, oeMCOLN1, oeNC + Era, and oeMCOLN1 + Era. Cells were incubated for an additional 24 h. Subsequently, cell viability and ferroptosis‐related indicators were evaluated.

### 2.4 Ferrostatin‐1 (Fer‐1) and Z‐VAD‐FMK Intervention

To further verify whether MCOLN1 depletion‐induced cell death was associated with ferroptosis or apoptosis, rescue experiments using ferroptosis and apoptosis inhibitors were performed. AGS and HGC27 cells were transfected with siNC or siMCOLN1 as described above. At 24 h after transfection, Fer‐1 (2 μM, MedChemExpress, China), a ferroptosis inhibitor, or Z‐VAD‐FMK (10 μM, MedChemExpress, China), a pan‐caspase apoptosis inhibitor, was added to the cells. The cells were divided into the following groups: siNC, siMCOLN1, siMCOLN1 + Fer‐1, and siMCOLN1 + Z‐VAD‐FMK. After an additional 24 h of treatment, cell viability and ferroptosis‐related indicators were measured.

### 2.5 Immunoblotting

RIPA lysis buffer (Servicebio, China) was used to lyse the cells. The quantified cell extracts were subjected to 4%–20% sodium dodecyl sulfate–polyacrylamide gel electrophoresis (Servicebio, China). The protein was subsequently electrically transferred to a polyvinylidene fluoride membrane (Servicebio, China). Five percent skim milk (Servicebio, China) was used to block the membrane. Afterward, the membrane was immersed in diluted primary antibodies overnight. Primary antibodies against MCOLN1 (1:1000 dilution; Proteintech, China), SLC7A11 (1:5000 dilution; Proteintech, China), ACSL4 (1:2000 dilution; Affinity Biosciences, China), AKT (1:2000 dilution; Affinity Biosciences, China), p‐AKT (1:2000 dilution; Affinity Biosciences, China), and GAPDH (1:2000 dilution; Servicebio, China) were used. Next, the membrane was incubated with an HRP‐linked secondary antibody at a 1:10000 dilution (Affinity Biosciences, China). The signal was visualized via an enhanced chemiluminescence (ECL) reagent (Meilunbio, China) and analyzed by ImageJ software (NIH, USA).

### 2.6 Cell Viability

Cell viability was assessed using the Cell Counting Kit‐8 (CCK‐8) reagent (Servicebio, China) following the manufacturer's protocol. In brief, cells seeded in 96‐well plates were incubated with CCK‐8 reagent for 2 h. The absorbance at 450 nm was then measured with a microplate reader (Huadong‐Electronics, China).

### 2.7 Malondialdehyde (MDA) and Glutathione (GSH) Assessment

Cells were lysed on ice, and the supernatant was collected for measurement of MDA and GSH levels. Commercial kits were used for this purpose: the MDA content was determined using an MDA Assay Kit (Servicebio, China), and the GSH level was measured with a Reduced GSH Assay Kit (Nanjing Jiancheng Bioengineering Institution, China). All procedures followed the respective kit instructions.

### 2.8 Reactive Oxygen Species (ROS) Evaluation

Intracellular ROS levels were evaluated using an ROS Assay Kit (Servicebio, China). Briefly, we prepared a working solution containing the DCFH‐DA probe and incubated the cells in it for 30 min. Images were then captured under an inverted fluorescence microscope (Motic, China), and fluorescence intensity was analyzed with ImageJ software. Relative intensity was calculated by setting the intensity of the control group (blank or siNC) to 1.

### 2.9 Intracellular Fe^2+^Measurement

Intracellular Fe^2+^ levels were measured using a FerroOrange Detection Kit (Servicebio, China). Briefly, the cells were stained with FerroOrange working solution for 30 min. After the residual probe was removed, the cells were observed under a fluorescence microscope, and images were captured. The integrated fluorescence intensity was then quantified with ImageJ software, and the relative intensity was calculated by normalization to that of the control group (blank or siNC), which was set to 1.

### 2.10 Co‐Immunoprecipitation (Co‐IP)

Co‐IP was performed to investigate the potential interaction between MCOLN1 and AKT. Briefly, AGS and HGC27 cells were harvested and lysed in IP lysis buffer (Beyotime, China). The lysates were collected and incubated with Protein A/G magnetic beads pre‐conjugated with the anti‐MCOLN1 antibody (Santa Cruz, USA) or normal IgG (Proteintech, China) at 4°C overnight with gentle rotation. The beads were washed several times with IP lysis buffer to remove nonspecifically bound proteins. The immunoprecipitated proteins were eluted and subsequently subjected to immunoblotting. The membranes were probed with antibodies against AKT and MCOLN1. A portion of the total cell lysate was retained as the input control, while normal IgG immunoprecipitation was used as the negative control.

### 2.11 Public Database Analyses

The protein–protein interaction (PPI) network was constructed on the basis of the STRING database [15]. The correlations among MCOLN1, TPCN1, TRPC1, and AKT1 expression were analyzed with the Gene Expression Profiling Interactive Analysis (GEPIA) database and are presented as Pearson's correlation coefficients [16].

### 2.12 Statistical Analysis

All the data are presented as the mean ± standard deviation (SD). Statistical analyses and graph generation were performed using GraphPad Prism 10 (Dotmatics, USA). Group comparisons were carried out by one‐way analysis of variance (ANOVA) followed by Tukey's post hoc test. A significance threshold of *p* < 0.05 was adopted.

## 3 Results

### 3.1 MCOLN1 Depletion Provoked Gastric Cancer Cell Ferroptosis

MCOLN1 depletion efficiency was determined by the decreased expression of MCOLN1 in AGS and HGC27 cells (Figure 1A). In both AGS and HGC27 cells, the level of MDA was elevated (Figure 2A), the level of GSH was reduced (Figure 2B), and the levels of ROS and Fe^2+^ were increased (Figure 2C,D) after MCOLN1 depletion. In addition, SLC7A11 expression was downregulated, but ACSL4 expression was not significantly changed in AGS cells after MCOLN1 depletion; in HGC27 cells, a lower level of SLC7A11 and a higher level of ACSL4 were observed after MCOLN1 depletion (Figure 3). Cell viability was reduced in AGS and HGC27 cells after MCOLN1 depletion (Figure S1). To further validate whether the observed cell death was ferroptosis, Fer‐1 (ferroptosis inhibitor) and Z‐VAD‐FMK (apoptosis inhibitor) were used. Fer‐1 intervention partially reversed the effects of MCOLN1 depletion on cell viability, MDA, and ROS in both AGS and HGC27 cells; however, Z‐VAD‐FMK did not alter the effects of MCOLN1 depletion on these parameters in either cell line (Figure S2A–C). These findings further supported the involvement of ferroptosis in MCOLN1 depletion‐induced cell death.

**FIGURE 1:**
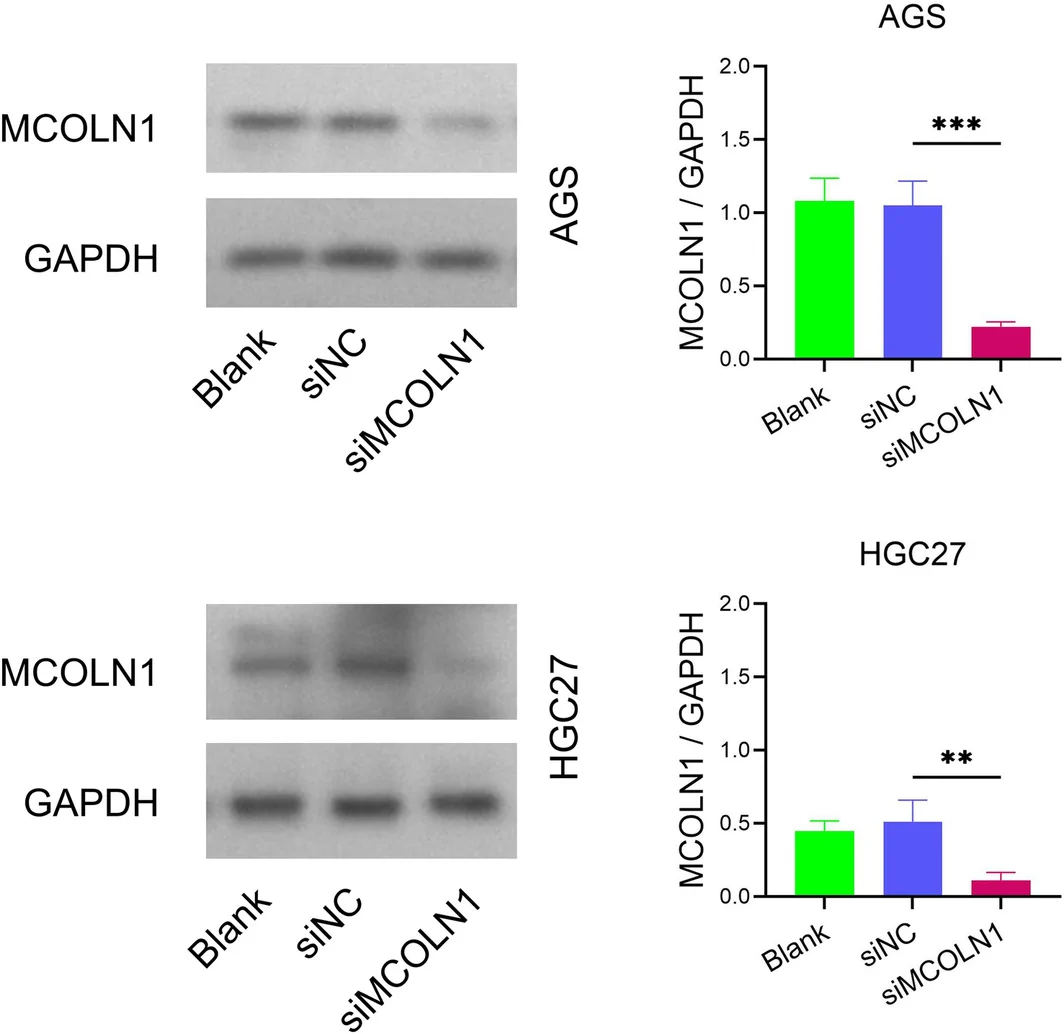
MCOLN1 depletion. MCOLN1 expression among the blank, siNC, and siMCOLN1 groups in gastric cancer cells. MCOLN1 expression was assessed by immunoblotting. Data were presented as mean ± SD. Three independent biological replicates were performed for each experiment. Statistical comparisons were performed using one‐way ANOVA followed by Tukey's post hoc test.

**FIGURE 2:**
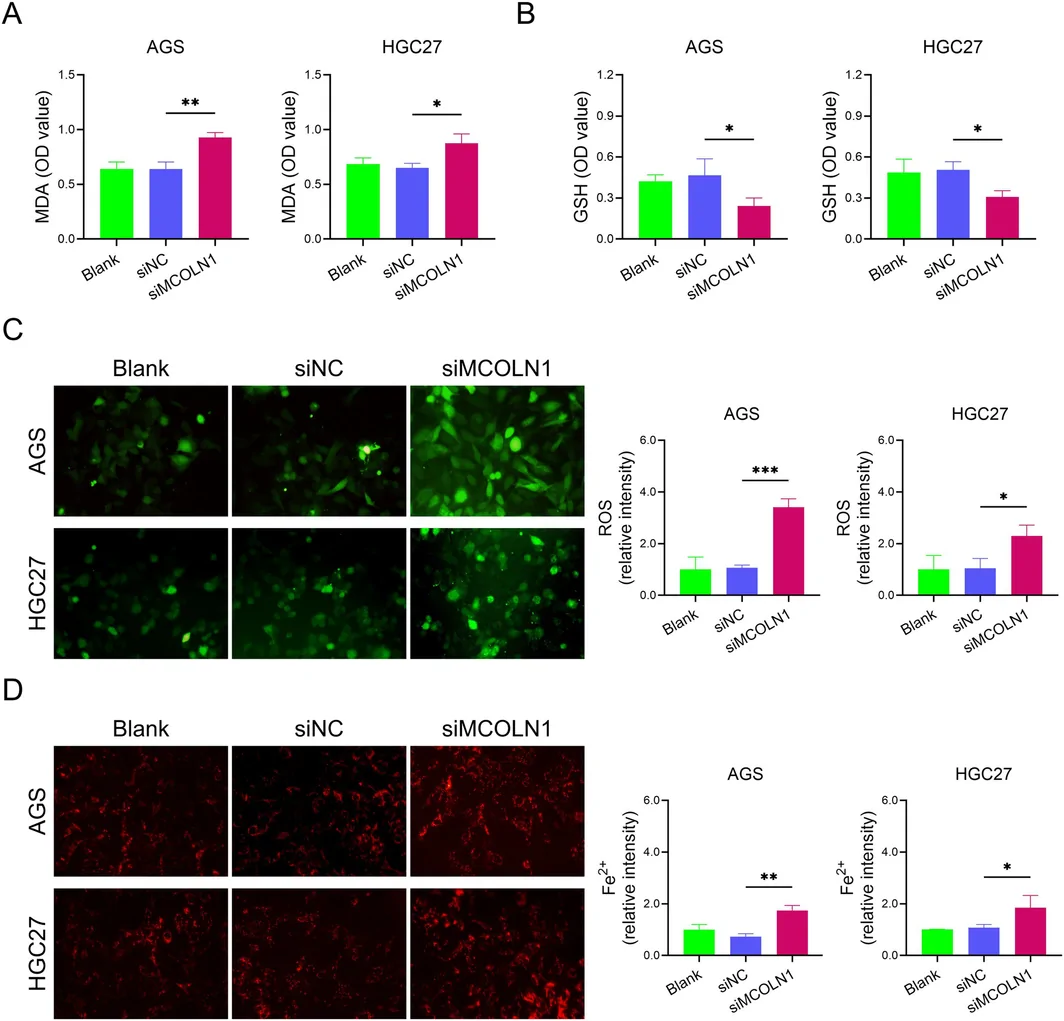
MCOLN1 depletion on ferroptosis markers. MDA (A), GSH (B), ROS (C), and Fe^2+^ (D) among the blank, siNC, and siMCOLN1 groups in gastric cancer cells. MDA and GSH levels were measured using commercial assay kits, while ROS and Fe^2+^ levels were evaluated using DCFH‐DA and FerroOrange staining, respectively. Data were presented as mean ± SD. Three independent biological replicates were performed for each experiment. Statistical comparisons were performed using one‐way ANOVA followed by Tukey's post hoc test.

**FIGURE 3:**
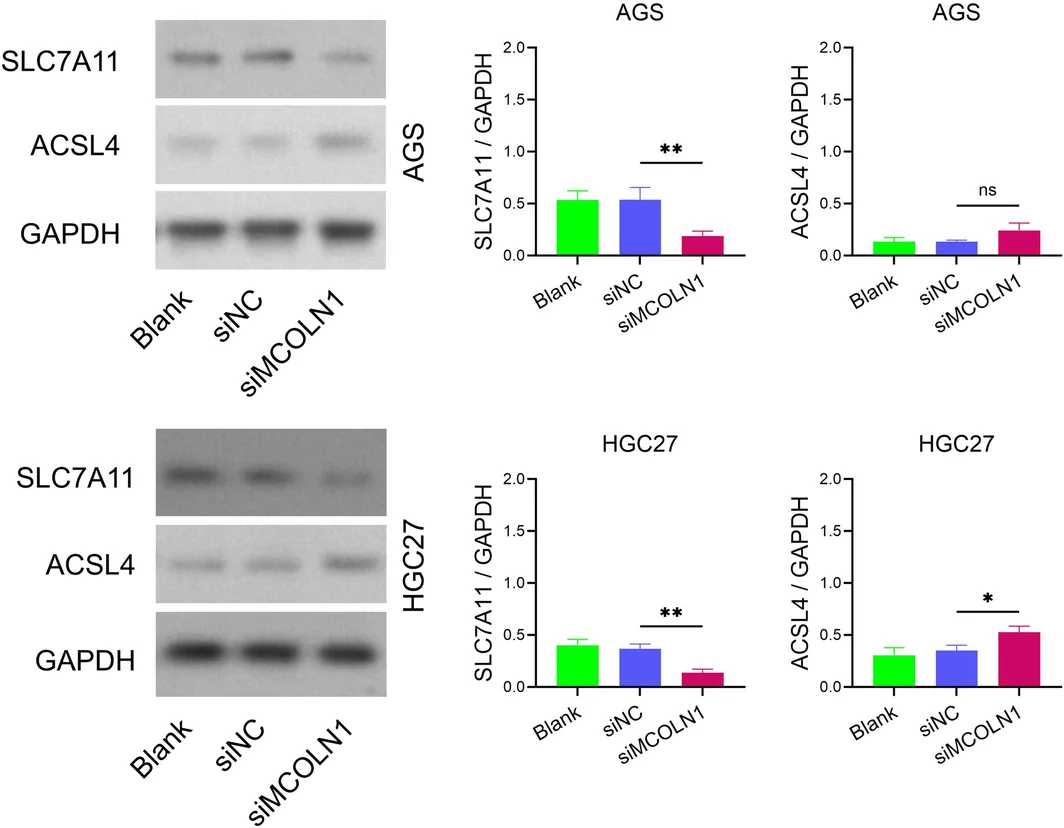
MCOLN1 depletion on other ferroptosis‐related protein markers. SLC7A11 and ACSL4 expression among the blank, siNC, and siMCOLN1 groups in gastric cancer cells. SLC7A11 and ACSL4 expression were assessed by immunoblotting. Data were presented as mean ± SD. Three independent biological replicates were performed for each experiment. Statistical comparisons were performed using one‐way ANOVA followed by Tukey's post hoc test.

### 3.2 Validation of the Role of MCOLN1 in Ferroptosis by MCOLN1 Overexpression and Era Intervention

The efficiency of MCOLN1 overexpression was confirmed by the increased MCOLN1 expression in both AGS and HGC27 cells (Figure S3A). Additionally, MCOLN1 overexpression increased cell viability in both AGS and HGC27 cells. In AGS cells, MCOLN1 overexpression reduced MDA and ROS levels, whereas only a decreasing trend was observed in HGC27 cells without statistical significance. Moreover, the ferroptosis inducer (Era) was used to further verify the effect of MCOLN1 overexpression on ferroptosis. Era intervention attenuated the effects of MCOLN1 overexpression on cell viability, MDA, and ROS in AGS and HGC27 cells (Figure S3B–D).

### 3.3 Molecular Regulatory Crosstalk Between MCOLN1 and AKT

The STRING‐based PPI network showed potential associations among MCOLN1, TRPC1, TPCN1, and AKT1, with TRPC1 and TPCN1 positioned between MCOLN1 and AKT1 in the network (Figure 4A). Correlation analyses of the GEPIA database revealed that MCOLN1 expression was positively correlated with TRPC1, TPCN1, and AKT1 expression in gastric cancer tissue samples (Figure 4B). Furthermore, in both AGS and HGC27 cells, p‐AKT was downregulated after MCOLN1 depletion (Figure 5). To further investigate how MCOLN1 regulated the AKT pathway Co‐IP was performed. AKT was detected in the immunoprecipitated complex obtained using an anti‐MCOLN1 antibody in both AGS and HGC27 cells, whereas no comparable signal was detected in the IgG control, supporting a potential physical association between MCOLN1 and AKT (Figure S4).

**FIGURE 4:**
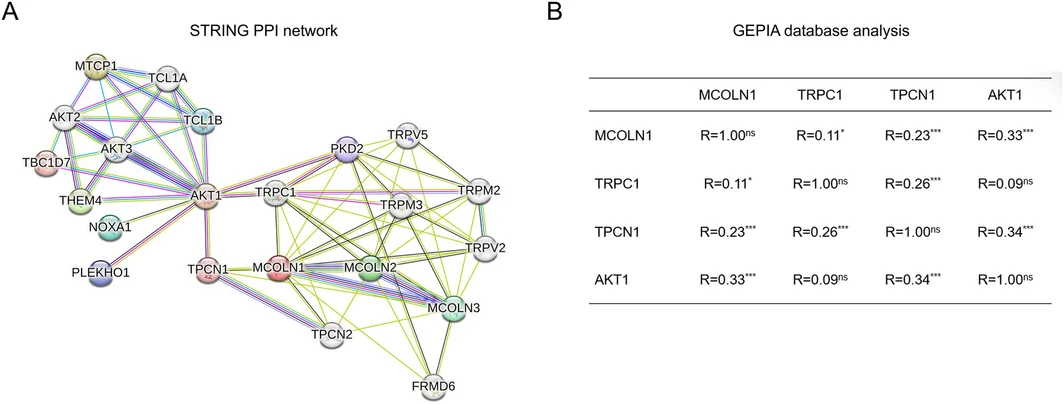
Relationship between MCOLN1 and AKT. PPI network (A) and correlation analyses (B) revealing connections among MCOLN1, TRPC1, TPCN1, and AKT1. The PPI network was constructed using the STRING database, and gene expression correlations were analyzed using the GEPIA database. Pearson's correlation coefficients were used to assess the correlations among these genes.

**FIGURE 5:**
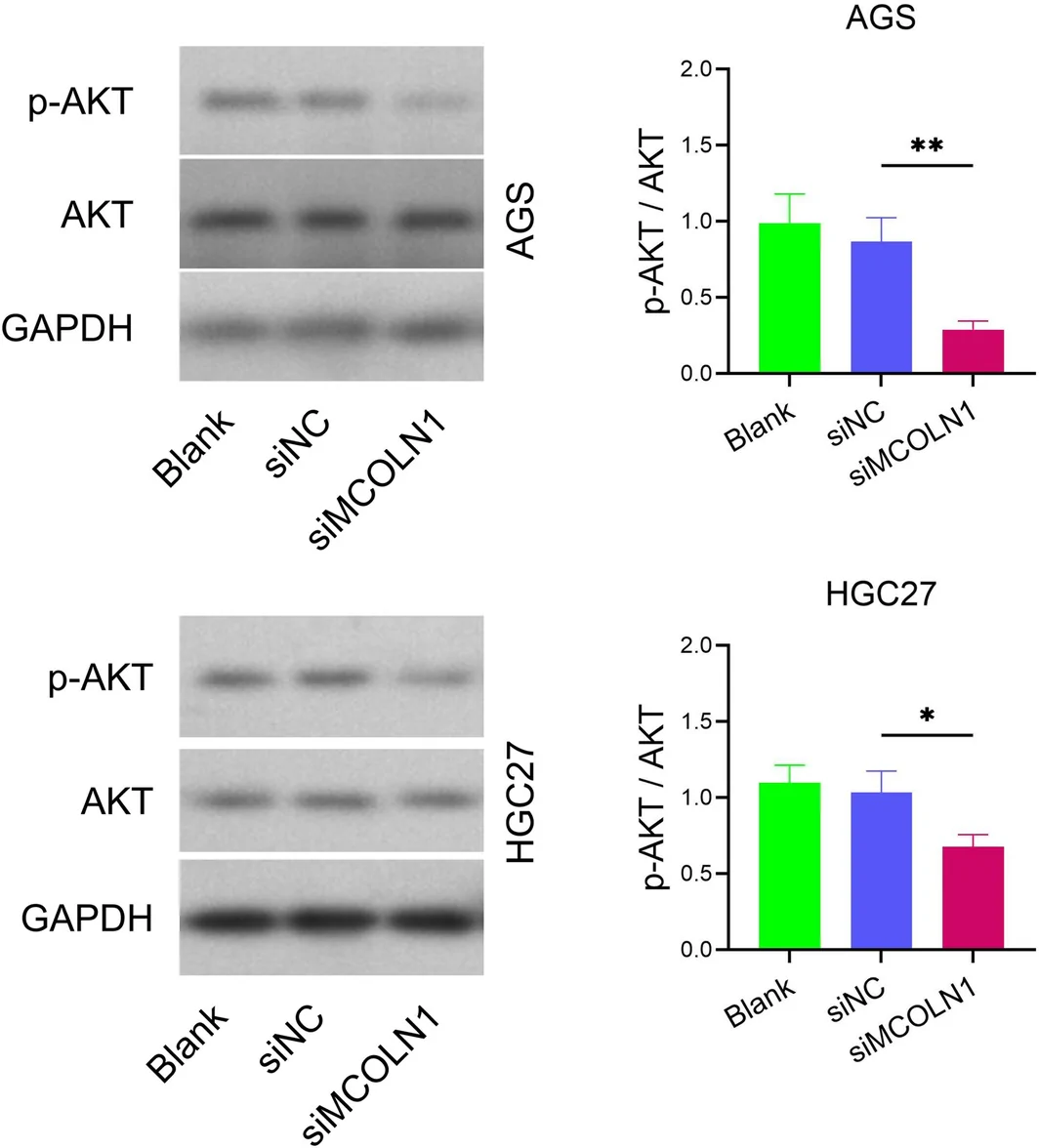
MCOLN1 depletion on the AKT pathway. p‐AKT/AKT expression among the blank, siNC, and siMCOLN1 groups in gastric cancer cells. p‐AKT and AKT expression were assessed by immunoblotting. Data were presented as mean ± SD. Three independent biological replicates were performed for each experiment. Statistical comparisons were performed using one‐way ANOVA followed by Tukey's post hoc test.

### 3.4 AKT Activation Attenuated the Regulatory Effect of MCOLN1 Depletion on Gastric Cancer Cell Ferroptosis

After SC79 intervention in AGS and HGC27 cells, p‐AKT was upregulated, and the regulatory effect of MCOLN1 depletion on p‐AKT was neutralized (Figure 6). MDA levels were lower (Figure 7A), GSH levels were higher (Figure 7B), and the levels of ROS and Fe^2+^ were lower (Figure 7C,D) after SC79 intervention in AGS and HGC27 cells, and the regulatory effect of MCOLN1 depletion on these parameters was reversed after SC79 intervention. Moreover, in AGS and HGC27 cells, SLC7A11 was upregulated but ACSL4 was downregulated after SC79 intervention, and the regulatory effect of MCOLN1 depletion on SLC7A11 and ACSL4 was also offset after SC79 intervention (Figure 8). Cell viability increased in AGS and HGC27 cells after SC79 intervention, and the regulatory effect of MCOLN1 depletion on cell viability was abolished after SC79 intervention (Figure S5).

**FIGURE 6:**
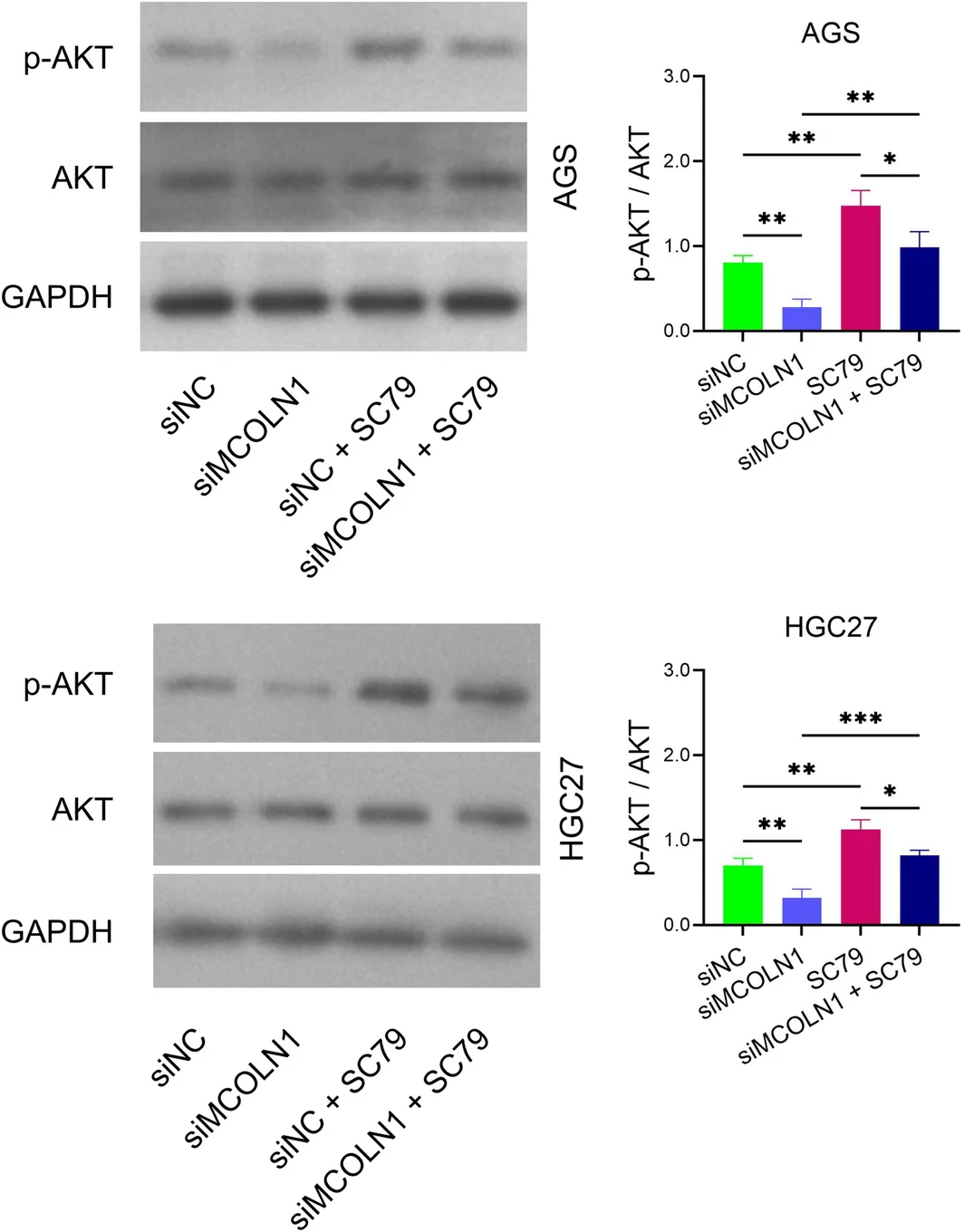
AKT pathway activation by SC79. p‐AKT/AKT expression among the siNC, siMCOLN1, SC79, and siMCOLN1 + SC79 groups in gastric cancer cells. p‐AKT and AKT expression was assessed by immunoblotting. Data were presented as mean ± SD. Three independent biological replicates were performed for each experiment. Statistical comparisons were performed using one‐way ANOVA followed by Tukey's post hoc test.

**FIGURE 7:**
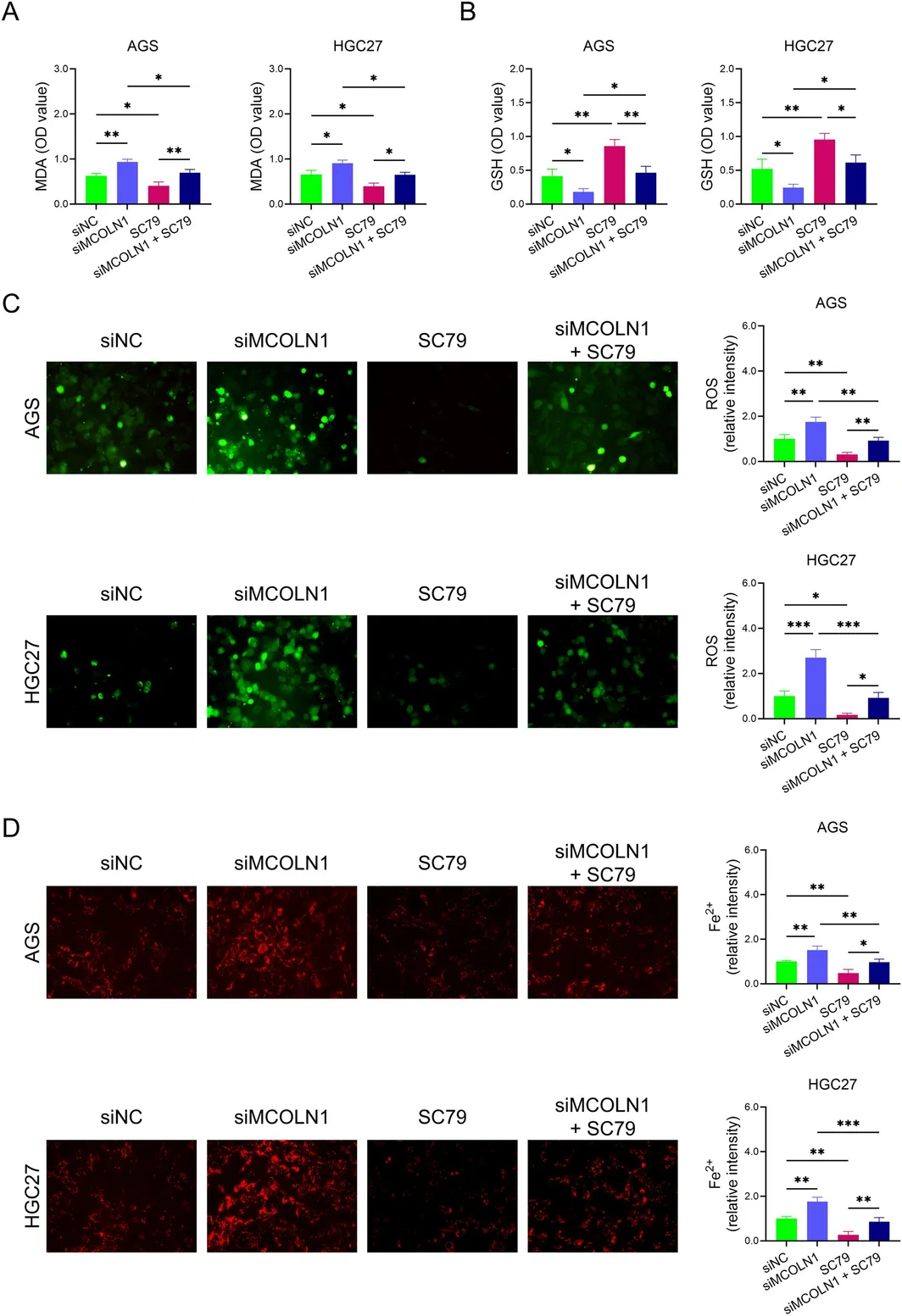
SC79 on ferroptosis markers. MDA (A), GSH (B), ROS (C), and Fe^2+^ (D) among the siNC, siMCOLN1, SC79, and siMCOLN1 + SC79 groups in gastric cancer cells. MDA and GSH levels were measured using commercial assay kits, while ROS and Fe^2+^ levels were evaluated using DCFH‐DA and FerroOrange staining, respectively. Data were presented as mean ± SD. Three independent biological replicates were performed for each experiment. Statistical comparisons were performed using one‐way ANOVA followed by Tukey's post hoc test.

**FIGURE 8:**
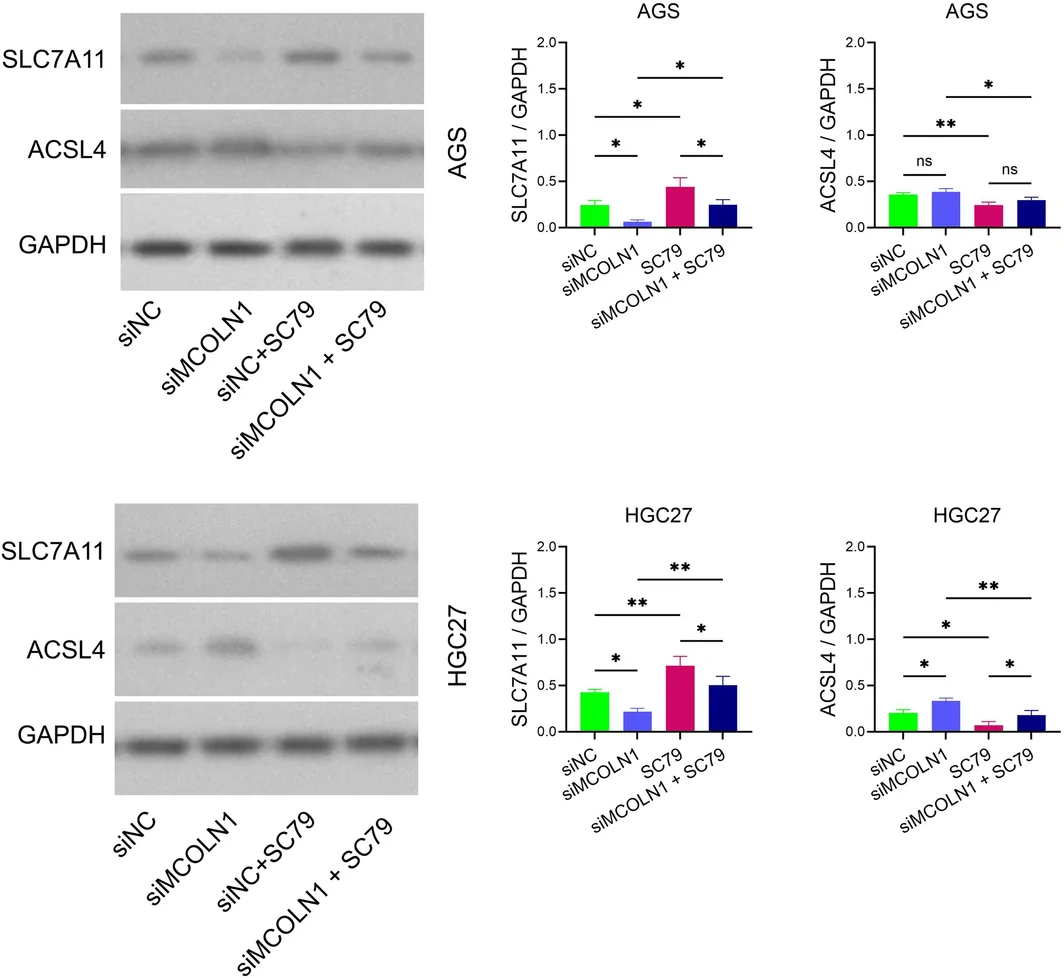
SC79 on other ferroptosis‐related protein markers. SLC7A11 and ACSL4 expression among the siNC, siMCOLN1, SC79, and siMCOLN1 + SC79 groups in gastric cancer cells. SLC7A11 and ACSL4 expression were assessed by immunoblotting. Data were presented as mean ± SD. Three independent biological replicates were performed for each experiment. Statistical comparisons were performed using one‐way ANOVA followed by Tukey's post hoc test.

## 4 Discussion

In recent years, as ferroptosis has garnered increasing attention as a novel form of cell death [17], MCOLN1 has also been reported to play an important role in inhibiting this process, with the most relevant studies published within the last 3 years [12, 13, 18, 19, 20, 21, 22]. Mechanistically, MCOLN1 coordinates with voltage‐dependent anion channel (VDAC) 3 to suppress ferroptosis in hepatocytes by regulating lysosome–mitochondria iron transfer [18]. MCOLN1 also exerts anti‐ferroptotic effects either directly or indirectly via the GPER pathway in atherosclerotic vascular endothelial cells [19]. Additionally, MCOLN1 has antiferroptotic activity in the context of antimony‐induced nephrotoxicity and during inflammatory responses to myocardial infarction [20, 21]. With respect to ferroptosis regulation in malignancies, MCOLN1 depletion has been reported to promote ferroptosis in breast cancer and hepatocellular carcinoma, and its regulatory effect on ferroptosis is more pronounced in cancers with AKT hyperactivation [12, 13, 22]. In the present study, we revealed that MCOLN1 depletion provoked gastric cancer cell ferroptosis. These findings were validated and explored from multiple dimensions, including the use of ROS, MDA, Fe^2+^, cell viability, and other ferroptosis biomarkers. The proferroptotic effect of MCOLN1 depletion in gastric cancer is consistent with the trends observed in other disease contexts [12, 13, 18, 19, 20, 21, 22]. On the basis of the literature and our subsequent investigations, we propose several potential mechanisms through which MCOLN1 regulates ferroptosis. First, MCOLN1 depletion may promote ferroptosis through inhibition of the AKT pathway, as supported by our follow‐up data. Second, it may regulate lysosome–mitochondrion iron transfer via interactions with VDAC1 or VDAC3 or through the GPER pathway to regulate ferroptosis [18, 19, 21]. Third, it may modulate chaperone‐mediated autophagy or zinc influx‐mediated autophagy to modify ferroptosis [20, 23].

MCOLN1 can activate the AKT pathway to accelerate tumor progression in gastric cancer [11] and prevent ferroptosis in AKT‐hyperactivated cancers [13]. These two studies suggest a possible association between MCOLN1 and AKT. Therefore, in the present study, we further explored this association and its potential mechanisms. The STRING‐based PPI network showed potential associations of MCOLN1 with AKT1, TRPC1, and TPCN1, while GEPIA analysis showed positive correlations between MCOLN1 and TRPC1, TPCN1, and AKT1 in gastric cancer tissue samples. Moreover, MCOLN1 depletion reduced p‐AKT levels in gastric cancer cells. These findings suggest that MCOLN1 may be involved in the regulation of the AKT pathway, with TRPC1 and TPCN1 potentially contributing to this process. TRPC1, a plasma membrane calcium‐permeable channel, contributes to store‐operated calcium entry and regulates intracellular calcium homeostasis, cellular proliferation, and migration [24]. TPCN1 is an intracellular cation channel predominantly localized to endolysosomal membranes and participates in the regulation of intracellular calcium and sodium signaling, endolysosomal function, and autophagy [25]. Of note, previous studies have shown that TRPC1 can regulate the AKT pathway through interactions with calmodulin (CaM) and the phosphatidylinositol 3‐kinase (PI3K) p85 subunit [26, 27], while TPCN1 has also been implicated in the AKT pathway through endolysosomal Ca^2+^ signaling [28]. Based on the above evidence, we speculated that TRPC1 and TPCN1 may mediate, at least in part, the effects of MCOLN1 on the AKT pathway by regulating ion homeostasis and related intracellular signaling processes. However, the precise roles of TRPC1 and TPCN1 in the MCOLN1‐regulated AKT pathway remain to be experimentally determined.

The AKT pathway is a classic cancer‐related signaling pathway. It regulates tumor growth, metastasis, and drug resistance and plays a role in ferroptosis [29, 30]. In gastric cancer, several studies have shown that activating the AKT pathway suppresses ferroptosis [31, 32, 33]. SC79 is a small molecule that selectively activates AKT. It binds to the PH domain of AKT, causing a conformational change that makes AKT more easily phosphorylated by upstream kinases, thereby activating the pathway [34]. In cancer research, SC79 is widely used for this purpose, as it is stable and strongly activated [35, 36, 37]. In the present study, we used SC79 to activate AKT in gastric cancer cells. Our results revealed that AKT activation inhibits ferroptosis in these cells, which is consistent with earlier reports [31, 32, 33]. Moreover, activating AKT weakened the ferroptosis‐promoting effect caused by MCOLN1 depletion. On the basis of the previous section on how MCOLN1 and AKT interact, we propose that MCOLN1 depletion promotes ferroptosis at least in part by inhibiting the AKT pathway.

This study revealed a cell line‐dependent difference in ACSL4 expression: MCOLN1 depletion increased ACSL4 expression in HGC27 cells but not in AGS cells. This discrepancy may be related to their distinct biological characteristics, as AGS cells are moderately to poorly differentiated gastric adenocarcinoma cells with epithelial‐like characteristics, whereas HGC27 cells are undifferentiated cancer cells with mesenchymal‐like features. Given the reported association between the mesenchymal state and ferroptosis sensitivity, HGC27 cells may rely more on ACSL4‐mediated lipid peroxidation for ferroptosis regulation, whereas AGS cells may involve ACSL4‐independent mechanisms [38, 39].

In the present study, we used SC79, a commonly used pharmacological AKT activator, to functionally reactivate AKT following MCOLN1 depletion. The attenuation of the ferroptosis‐related effects induced by MCOLN1 depletion following SC79 treatment provides supportive evidence for the involvement of the AKT pathway. However, genetic rescue experiments, such as MCOLN1 overexpression in knockdown cells or constitutively active AKT, would further strengthen the causal relationship between MCOLN1 and the AKT pathway. Further studies should be conducted to validate this proposed regulatory relationship through genetic rescue experiments. In addition, validation in clinical gastric cancer specimens would provide more direct evidence regarding the clinical relevance of MCOLN1 in gastric cancer. Therefore, further clinical studies are warranted to investigate the association of MCOLN1 expression with the prognosis of gastric cancer patients. Moreover, multiple levels of MCOLN1 were not experimentally examined in the present study, and thus a strict dose‐dependent relationship between MCOLN1 expression and ferroptosis resistance could not be established. Future studies are warranted to further investigate the dose‐dependent effects of MCOLN1 on ferroptosis.

## 5 Conclusion

In conclusion, MCOLN1 depletion promotes gastric cancer cell ferroptosis, at least in part, by inhibiting the AKT pathway. TRPC1 and TPCN1 may potentially contribute to the association between MCOLN1 and the AKT pathway, but their specific roles require further investigation. Our study suggests that MCOLN1 may be a potential ferroptosis‐related target for gastric cancer treatment, but more substantial and in‐depth studies are needed to further verify this finding.

## Conflicts of Interest

The authors declare no conflicts of interest.

## Supporting information


**Figure S1:** MCOLN1 depletion on cell viability. Cell viability among the blank, siNC, and siMCOLN1 groups in gastric cancer cells. Cell viability was assessed using the CCK‐8 assay. Data were presented as mean ± SD. Three independent biological replicates were performed for each experiment. Statistical comparisons were performed using one‐way ANOVA followed by Tukey's post hoc test.

**Figure S2:** Validation of MCOLN1 depletion‐induced ferroptosis. Cell viability (A), MDA (B), and ROS (C) levels among the siNC, siMCOLN1, siMCOLN1 + Fer‐1, and siMCOLN1 + Z‐VAD‐FMK groups in gastric cancer cells. Cell viability was assessed using the CCK‐8 assay, MDA levels were measured using an MDA assay kit, and ROS levels were evaluated using DCFH‐DA staining. Data were presented as mean ± SD. Three independent biological replicates were performed for each experiment. Statistical comparisons were performed using one‐way ANOVA followed by Tukey's post hoc test.

**Figure S3:** Validation of the role of MCOLN1 in ferroptosis. MCOLN1 expression (A), cell viability (B), MDA (C), and ROS (D) levels among the oeNC, oeMCOLN1, oeNC + Era, and oeMCOLN1 + Era groups in gastric cancer cells. MCOLN1 expression was assessed by immunoblotting, cell viability was assessed using the CCK‐8 assay, MDA levels were measured using an MDA assay kit, and ROS levels were evaluated using DCFH‐DA staining. Data were presented as mean ± SD. Three independent biological replicates were performed for each experiment. Statistical comparisons were performed using one‐way ANOVA followed by Tukey's post hoc test.

**Figure S4:** Interaction between MCOLN1 and AKT. Co‐IP analysis of the potential interaction between MCOLN1 and AKT in gastric cancer cells. The potential interaction between MCOLN1 and AKT was assessed by Co‐IP followed by immunoblotting. Three independent biological replicates were performed for each experiment.

**Figure S5:** SC79 on cell viability. Cell viability among the siNC, siMCOLN1, SC79, and siMCOLN1 + SC79 groups in gastric cancer cells. Cell viability was assessed using the CCK‐8 assay. Data were presented as mean ± SD. Three independent biological replicates were performed for each experiment. Statistical comparisons were performed using one‐way ANOVA followed by Tukey's post hoc test.

## Data Availability

The data that support the findings of this study are available from the corresponding author upon reasonable request.
